# Association Between Antidepressant Use and Risk of Venous Thromboembolism: A Systematic Review and Meta-Analysis

**DOI:** 10.3390/jcm14155512

**Published:** 2025-08-05

**Authors:** Minyoung Uh, Hey Young Rhee, Kiyon Rhew

**Affiliations:** 1 College of Pharmacy, Dongduk Women’s University, Seoul 02748, Republic of Korea; 20234008@dongduk.ac.kr; 2 Division of Library & Information Science, College of Social Sciences, Dongduk Women’s University, Seoul 02748, Republic of Korea; jonju@dongduk.ac.kr

**Keywords:** antidepressants, venous thromboembolism, deep vein thrombosis, pulmonary embolism, meta-analysis, systematic review

## Abstract

**Objectives**: To evaluate the association between antidepressant use and the risk of venous thromboembolism (VTE), including deep vein thrombosis and pulmonary embolism (PE), through a systematic review and meta-analysis of observational studies. **Methods**: A comprehensive literature search was conducted in Medline, Embase^®^, and Web of Science^®^ up to December 2024. Eighteen studies (cohort, case-control, and nested case-control designs) meeting inclusion criteria were analyzed. Study quality was assessed using the Newcastle–Ottawa Scale. Pooled relative risks (RR) with 95% confidence intervals (CIs) were calculated using a random-effects model. Subgroup analyses were performed based on recency of antidepressant use, VTE onset type (first vs. recurrent), and VTE subtype (PE). **Results**: Antidepressant use was associated with a significantly increased risk of VTE (RR = 1.22; 95% CI: 1.12–1.32; *p* < 0.001). Subgroup analyses revealed a stronger association for recent use (within 90 days), first-onset VTE, recurrent VTE, and PE. Heterogeneity was high (I^2^ = 87.92%), but sensitivity analysis confirmed result robustness. No publication bias was detected. **Conclusions**: This meta-analysis indicates a modest but statistically significant increase in the risk of VTE associated with antidepressant use, particularly among recent users, individuals experiencing either first-time or recurrent VTE, and those with PE-type events. These findings highlight the importance of individualized VTE risk assessment when initiating antidepressant therapy.

## 1. Introduction

Venous thromboembolism (VTE) is a condition in which a thrombus—a blood clot formed within the venous circulation—travels and obstructs a blood vessel, resulting in an embolism [1,2]. Depending on the site of occurrence, VTE can be classified as either deep vein thrombosis (DVT) or pulmonary embolism (PE) [2]. Virchow’s triad describes the most well-known causes of thrombus formation: venous stasis, hypercoagulability, and endothelial injury. These factors may act independently or in combination with one another [3].

Various risk factors contribute to the development of VTE, including lower limb fractures, joint surgeries, malignancy, pregnancy, use of oral contraceptives, prolonged immobility, and long-distance air travel [4,5,6,7,8,9,10,11,12]. Importantly, VTE can also be triggered by conditions commonly encountered in daily life, not only by severe illnesses [13]. Chronic cardiovascular conditions such as obesity, hypertension, and dyslipidemia have also been identified as significant risk factors [14,15].

In addition to the well-established factors mentioned above, recent studies have investigated the potential association between psychotropic medications, particularly antipsychotics and antidepressants, and the risk of VTE [16,17,18]. Two meta-analyses have suggested that the use of antidepressants may be associated with an increased risk of VTE [19,20]. A prior meta-analysis reported a significant association between antidepressant use and VTE using a random-effects model; however, as noted by Rhew et al., fixed-effects models were applied in certain subgroup analyses, resulting in inconsistent effect estimates and raising concerns about interpretational reliability [21]. Another meta-analysis published in 2019 found a statistically significant association overall; however, this association was not observed in a subgroup analysis that included only studies with a low risk of bias [20].

Given that antidepressants are widely prescribed not only for depression but also for conditions such as chronic pain, generalized anxiety disorder, obsessive-compulsive disorder, and eating disorders, a large number of patients are exposed to these medications [22,23]. Venous thromboembolism affects approximately 1–2 per 1000 individuals annually, particularly in older adults [24]. Antidepressant use has steadily increased worldwide, with more than 300 million users globally according to recent estimates [25]. Despite growing interest in this potential association, previous meta-analyses on the topic included studies only up to 2017–2018 and did not examine clinically important subgroups such as recurrent VTE or timing of antidepressant use. To address these gaps, we conducted an updated and comprehensive meta-analysis using newly available observational studies. This study aims to determine whether antidepressant use increases the risk of VTE through a systematic review and meta-analysis of observational data.

## 2. Materials and Methods

### 2.1. Search Strategy and Databases

We systematically searched Medline, Embase^®^, and Web of Science^®^ for relevant studies published up to December 2024. The search strategy included terms related to both the exposure and the outcome. Searching terminology was “antidepressant OR antidepressive OR “monoamine oxidase inhibitors” OR “selective serotonin reuptake inhibitors” OR “serotonin norepinephrine reuptake inhibitors” OR tricyclic OR SSRI OR SNRI OR TCA OR MAO) AND (thromboemboli* OR “venous thrombo*” OR “pulmonary emboli*” OR “deep vein thrombo*)””. Only studies involving human subjects were considered, and the systematic review was conducted according to the PRISMA (Preferred Reporting Items for Systematic Reviews and Meta-Analyses) guidelines.

### 2.2. Study Inclusion and Exclusion Criteria

We included studies that examined the association between antidepressant use and VTE, including PE and DVT. Duplicated results across databases were excluded, as were non-primary research articles such as case reports, review articles, and conference abstracts. Two independent reviewers (MU and HL) screened titles and abstracts, reviewed the full texts of potentially eligible studies, and independently extracted data and assessed study quality.

### 2.3. Data Extraction and Quality Assessment

Using a standardized form, we extracted key study characteristics, including population demographics, the country in which the study was conducted, total sample size, definitions of exposure and outcome, number of outcome events, covariates for adjusting groups, and types of antidepressants used. The primary outcome was defined as any VTE event (e.g., DVT, PE) in subjects without a prior history of significant VTE risk. Study quality was assessed using the Newcastle–Ottawa Scale (NOS), which evaluates observational studies across eight items grouped into three domains: selection of study groups, comparability between groups, and ascertainment of exposure and outcomes [26]. The scale assigns a maximum score of nine, indicating the lowest risk of bias. Discrepancies in quality assessment were resolved through discussion and consensus with a third reviewer (KR).

### 2.4. Statistical Analysis

We calculated pooled relative risks (RRs) with 95% confidence intervals (CIs) to estimate the association between antidepressant use and the risk of venous thromboembolism. When exact numbers of events and non-events were not reported, odds ratios (ORs) or hazard ratios (HRs) were treated as equivalent to RRs, as this approximation is considered appropriate for rare outcomes [27]. Heterogeneity across studies was assessed using the I^2^ statistic and Cochran’s Q test. A random-effects model was applied when the *p*-value for heterogeneity was <0.05. Sensitivity analysis was conducted using a leave-one-out approach, and publication bias was evaluated visually with funnel plots and statistically with Begg’s rank correlation and Egger’s regression tests.

Subgroup analyses were performed to explore potential sources of heterogeneity and to assess clinically relevant differences in risk estimates. Specifically, we conducted subgroup analyses by (1) recency of antidepressant use (within 90 days), (2) VTE onset type (first-onset vs. recurrent VTE), and (3) VTE subtype (PE). These analyses aimed to determine whether the association between antidepressant use and VTE risk varied according to exposure timing, clinical context, or outcome type.

Subgroup analyses were conducted based on stratified effect estimates reported in the original studies or derived from study-level information. Specifically, studies were included in each subgroup if they either (1) reported risk estimates by exposure timing, event type, or outcome subtype, or (2) provided sufficient data to classify the exposure or outcome according to our predefined criteria (e.g., studies describing antidepressant use within 90 days as “recent” or those exclusively focusing on first-onset events or PE). No imputation or reconstruction of effect sizes was performed.

All analyses were performed using Comprehensive Meta-Analysis software (version 4.0; Biostat, Englewood, NJ, USA). A two-sided *p*-value < 0.05 was considered statistically significant.

## 3. Results

### 3.1. Study Selection and Characteristics

**Figure 1:**
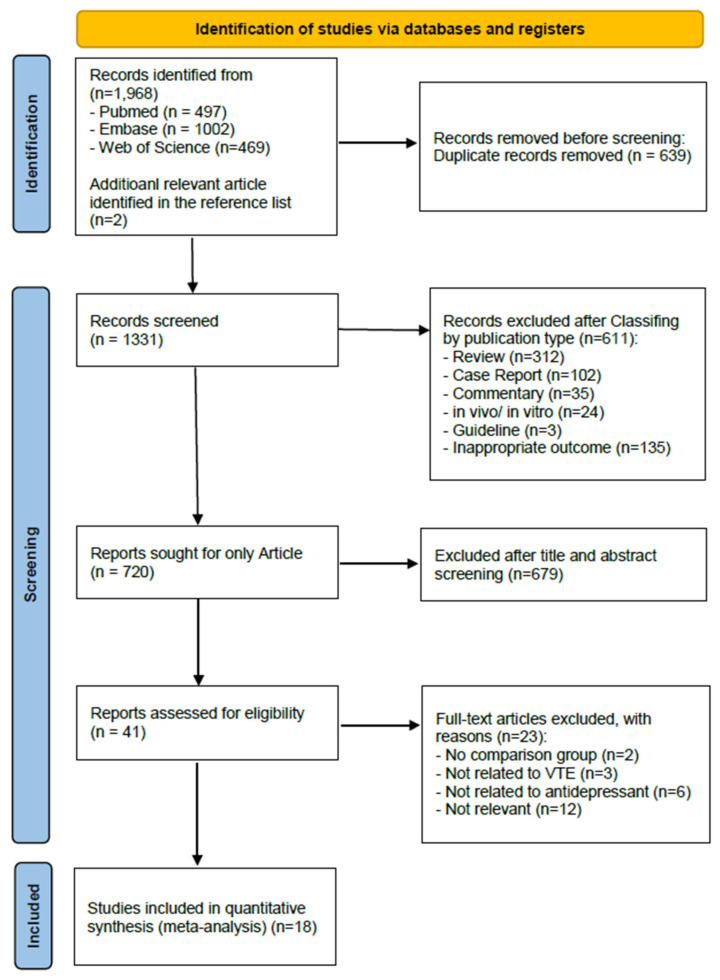
PRISMA flow diagram of the study selection process.

A total of 1968 records were identified through database searching, and 639 duplicates were removed. After screening 1331 titles and abstracts and assessing 41 full-text articles, 18 studies were included in the final meta-analysis (Figure 1) [28,29,30,31,32,33,34,35,36,37,38,39,40,41,42,43,44,45]. The included studies were published between 2000 and 2024 across 12 countries, with the United Kingdom, France, Denmark, and the United States being most represented. Study designs included cohort studies (n = 7), case-control/nested case-control (n = 10), and one case-crossover study. Sample sizes ranged from 214 to over 730,000, and the proportion of female participants varied from 49.5% to 100%. Most studies included adults aged ≥18 years, with several focusing on middle-aged or older populations. Regarding exposure, selective serotonin reuptake inhibitors (SSRIs) were the most commonly studied antidepressants, followed by tricyclic antidepressants (TCAs), serotonin–norepinephrine reuptake inhibitors (SNRIs), or combinations. Some studies reported specific agents such as sertraline or escitalopram. The study quality, assessed using the NOS, ranged from 7 to 9, indicating generally high methodological quality. Most studies adjusted for key covariates, including age, sex, comorbidities (e.g., cardiovascular disease, diabetes), lifestyle factors (e.g., smoking, body mass index), and concurrent medications (e.g., anticoagulants, hormone therapy) (Table 1). The quality of the included studies was assessed using the Newcastle–Ottawa Scale (NOS), and detailed scores are presented in Supplementary Table S1. To enhance transparency and address potential publication bias, we provide a detailed summary of data sources and collection methods for all included studies in Supplementary Table S2.

### 3.2. Association Between Antidepressants and VTE

**Figure 2:**
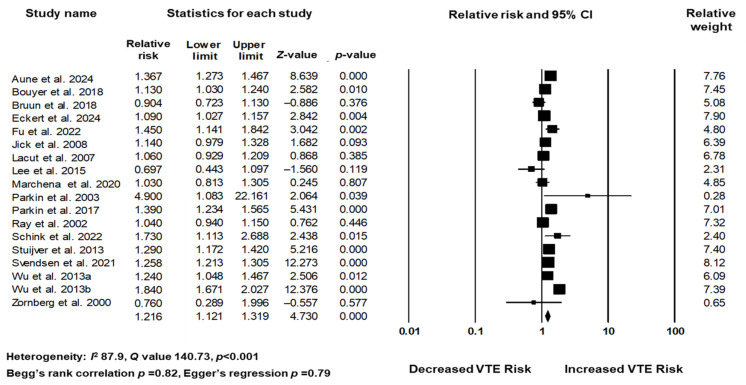
Associations of antidepressant use and risk of venous thromboembolism, based on studies [26,27,28,29,30,31,32,33,34,35,36,37,38,39,40,41,42,43].

The association between antidepressant use and the risk of VTE was quantitatively assessed using a meta-analysis of 18 studies. Individual study estimates for the RR ranged from 0.90 to 2.00. The pooled analysis revealed a statistically significant association, with an overall RR of 1.22 (95% CI: 1.12–1.32, *p* < 0.001), indicating a modest increase in VTE risk among antidepressant users. Significant heterogeneity was observed across studies (I^2^ = 87.92%, τ^2^ = 0.021), reflecting differences in study design, population characteristics, types of antidepressants evaluated, and analytical methods. Notably, several studies, such as those by Aune et al., Wu et al. (2013b) [42], and Zornberg et al. [43], reported higher risk estimates, whereas others, including Marchena et al. [36] and Parkin et al. (2003) [37], found little or no increased risk (Figure 2).

### 3.3. Subgroup Analysis

**Figure 3:**
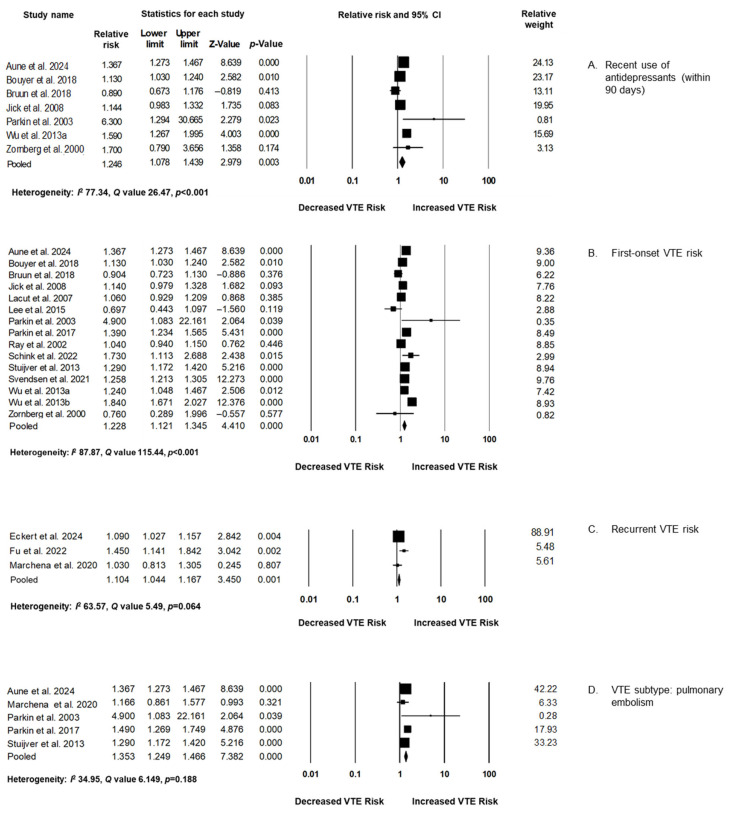
Subgroup analyses exploring the association between antidepressant use and venous thromboembolism, based on studies [26,27,28,29,30,31,32,33,34,35,36,37,38,39,40,41,42,43].

Subgroup analyses were performed to explore whether the association between antidepressant use and VTE risk varied by exposure timing, event type, or outcome subtype. A significantly increased risk of VTE was observed among recent users of antidepressants, defined as those with exposure within 90 days prior to the thrombotic event. This finding suggests a possible time-dependent relationship, whereby thromboembolic risk is elevated during the early period of treatment or recent use. The association was also notably stronger in studies that focused on first-onset VTE compared to those evaluating recurrent events. While the pooled effect estimate for recurrent VTE showed a modest increase, it was not statistically significant, indicating a differential risk pattern between incident and recurrent VTE cases. Furthermore, when the analysis was limited to studies reporting PE as the outcome, the effect size was more pronounced compared to analyses including all types of VTE. This suggests that PE may be a particularly sensitive outcome to the prothrombotic effects of antidepressants. Collectively, these subgroup findings reinforce the importance of considering patient history and timing of antidepressant exposure when assessing thromboembolic risk (Figure 3).

### 3.4. Sensitivity Analysis and Publication Bias

The sensitivity analysis showed no significant changes in the pooled estimate when each study was removed in turn, indicating the robustness of the overall results. Funnel plot inspection revealed a symmetrical distribution, and Begg’s test did not suggest the presence of publication bias. Full details, including forest plots, funnel plots, and NOS domain-level scores, are presented in the Supplementary Materials (Figure S1 and Figure S2).

## 4. Discussion

Our meta-analysis demonstrated a significant association between antidepressant use and an increased risk of VTE, including both DVT and PE (RR = 1.22, 95% CI: 1.12–1.32; *p* < 0.001). The pooled effect size across included studies indicated a modest but consistent elevation in VTE risk among antidepressant users compared to non-users or control groups. The findings of our meta-analysis are broadly consistent with those of previous systematic reviews that have suggested a modestly increased risk of VTE associated with antidepressant use. For instance, a 2018 meta-analysis by Kunutsor et al. and a more recent analysis by Wang et al. both reported pooled effect sizes indicating a statistically significant association [19,20]. However, those studies were limited by the relatively small number of included articles and restricted subgroup analyses.

In contrast, our study included a substantially larger number of eligible studies and a broader range of populations, allowing for more robust estimates and refined subgroup analyses. Furthermore, we were able to separately examine first-onset versus recurrent VTE, as well as outcome-specific differences such as PE and recent antidepressant use, which had not been adequately addressed in earlier work. These additions enhance the granularity and clinical applicability of our findings, supporting a more nuanced understanding of VTE risk patterns across different patient subgroups. By extending and refining prior analyses, our meta-analysis contributes more substantial evidence to guide clinical decision-making in patients initiating or continuing antidepressant therapy.

The elevated risk observed in patients with recent antidepressant use may reflect a temporally sensitive period during which pharmacologic and behavioral factors converge to increase thrombotic susceptibility. In our analysis, “recent use” was defined as antidepressant exposure within 90 days prior to the VTE event. This does not necessarily reflect treatment initiation, as exposure could include both new and ongoing users; nonetheless, it captures a period of close temporal proximity to thrombotic onset. While prior studies of other medication classes, such as oral contraceptives, hormone therapy using estrogen, and antipsychotics, have demonstrated that VTE risk is highest in the early phase after treatment initiation [46,47,48], no comparable evidence exists to date for antidepressants. The rarity of VTE, particularly in the general population, poses substantial challenges for conducting large-scale prospective studies to examine precise timing effects in this context. Consequently, our meta-analysis offers novel insight by quantifying VTE risk in relation to recent antidepressant exposure, a temporal association that has not been systematically addressed in previous research. Although our design does not allow determination of causality or differentiation between incident and prevalent users, the results nonetheless suggest that recency of antidepressant use may be a relevant factor in thrombotic risk assessment, particularly in high-risk populations.

Our meta-analysis also examined whether the association between antidepressant use and VTE differed by event type, particularly between first-onset and recurrent events. A statistically significant association was observed in both subgroups, with a numerically greater effect size in the first-onset VTE group. However, no formal statistical test was conducted to compare the subgroups directly. This finding suggests that antidepressant use may increase thromboembolic risk not only in new cases but also among patients with a prior history of VTE. This aligns with earlier findings, such as those by Lacut et al. (2007), which identified antidepressant exposure as a novel risk factor for initial VTE presentation [37]. In our updated subgroup analysis, however, a statistically significant association was also observed in studies assessing recurrent VTE, a result not consistently reported in previous meta-analyses. While this finding may suggest that antidepressant use poses thromboembolic risks beyond first-time events, interpretation must be approached with caution due to the limited number of contributing studies and potential variability in study designs. Several mechanisms may underlie this observed association. Patients with a history of VTE often have persistent or cumulative risk factors such as inherited thrombophilia, malignancy, or chronic inflammation [49,50,51]. The use of antidepressants in such patients may interact with these underlying risks to increase the likelihood of recurrence. Moreover, behavioral or medication-related changes following the initial VTE event, such as reduced mobility, polypharmacy, or incomplete adherence to thromboprophylaxis, could further contribute to risk [52,53,54]. Importantly, these findings challenge the prior assumption that antidepressants primarily act as a precipitating factor for first-onset events. Instead, our results indicate that patients with prior VTE may also be vulnerable, particularly if multiple risk factors coexist. Clinicians should, therefore, be mindful of antidepressant prescribing in this high-risk group and consider comprehensive risk-benefit evaluations when initiating or continuing therapy. Prospective studies with stratified risk assessment and mechanistic data are needed to confirm these associations and guide secondary prevention strategies.

When subgroup analysis was restricted to studies reporting PE as the outcome, the association with antidepressant use remained significant and showed a more pronounced effect size compared to general VTE or DVT-specific outcomes. This observation is notable, as PE represents a more severe and clinically apparent manifestation of VTE. Our results echo those of the study by Parkin et al. (2003), which found that SSRI and TCA use was associated with a higher risk of PE hospitalization, even after adjusting for depression severity and immobility [39]. One possible explanation is that the hemodynamic and vascular effects of certain antidepressants may disproportionately affect the pulmonary vasculature [55,56]. Alternatively, because PE is more likely to be investigated and diagnosed due to its acute presentation, there may be a detection bias favoring its identification in patients under regular medical surveillance, such as those receiving psychiatric treatment [57,58,59]. Nonetheless, the consistency of the observed association across studies focusing on PE strengthens the plausibility of a class-related thromboembolic risk, particularly for PE, and suggests that this outcome deserves further targeted research.

While other potentially relevant moderating factors, such as antidepressant class, sex, or comorbid conditions, could affect the risk of VTE, subgroup analyses were limited to variables with sufficient data reported across studies. We deliberately prioritized moderators with both clinical relevance and adequate data availability to ensure the robustness and interpretability of our findings. Specifically, we focused on recency of use, event type, and outcome subtype, which allowed us to preserve statistical power while capturing clinically meaningful differences in risk. This approach reflects a balance between analytical feasibility and practical utility, emphasizing results that may directly inform patient care and risk stratification strategies. In some cases, subgroup classification was based on study-reported stratified estimates; in others, we used clear operational definitions to categorize studies using descriptive or tabular data. This ensured consistency and transparency while allowing us to capture clinically relevant heterogeneity across studies.

This meta-analysis is not without limitations. First, most of the included studies were observational in nature, which introduces the possibility of residual confounding. Depression itself is associated with known VTE risk factors, such as immobility, systemic inflammation, and metabolic changes, which may not have been fully accounted for in all included studies. Additionally, many studies lacked detailed data on antidepressant dose, duration, or adherence, limiting the ability to assess dose-response relationships or temporal trends. There was also heterogeneity in the definitions and diagnostic criteria for VTE outcomes across studies, which may have contributed to variability in effect estimates. Third, although most studies adjusted for psychiatric comorbidities and lifestyle factors, residual confounding by indication cannot be ruled out. Depression itself may elevate VTE risk through mechanisms such as systemic inflammation, reduced physical activity, or other behavioral factors, which may not have been fully accounted for in all included studies. Lastly, although formal tests for publication bias were negative, the possibility of selective reporting cannot be completely excluded. Despite these limitations, the study has several important strengths. It includes the largest number of eligible studies to date examining the association between antidepressant use and VTE, enhancing statistical power and generalizability. In addition, our study incorporates recently published observational studies that were not available to earlier meta-analyses, providing timely and updated evidence on this association. The breadth of populations and settings improves the applicability of results to diverse clinical scenarios. Furthermore, this study is the first to comprehensively explore subgroup effects by exposure timing, event type (first vs. recurrent), and outcome (PE only), offering a more refined risk profile than previous analyses. The methodological rigor, including adherence to PRISMA guidelines and thorough quality assessment, strengthens the credibility of our findings and reinforces their clinical relevance.

## 5. Conclusions

This comprehensive meta-analysis found a statistically significant association between antidepressant use and increased risk of VTE, particularly among recent users, and in patients experiencing either a first or recurrent VTE event, including PE. These findings highlight the importance of incorporating VTE risk stratification into clinical decision-making when prescribing antidepressants, especially for individuals with additional thrombotic risk factors. Further prospective studies are warranted to clarify the causal relationship and elucidate the underlying biological mechanisms.

## Figures and Tables

**Table 1:** Characteristics of included studies.

Study	Country	Study Design	No. of Patients	Female (%)	Age	Types of Antidepressants	NOS	Covariate
Aune et al. (2024) [31]	Norway, Sweden	case-crossover	48,192	53.2	≥30	Antidepressant (sertraline, escitalopram, mianserin, and mirtazapine)	8	No covariate adjusted
Bouyer et al. (2018) [32]	France	cohort	323,737	48.6	≥18	Not Classified	8	Age, obesity, active cancer, previous thromboembolism, severe paralysis, renal disease, psychiatric disease, and use of OC
Bruun et al. (2018) [28]	Denmark	prospective cohort	68,487	71.2	≥18	SSRI	8	Age, gender, marital status, operation year, MI, CHF, PVD, dementia, chronic pulmonary disease, connective tissue disease, ulcer disease, LD, DM, hemiplegia, moderate to severe renal disease, cancer, NSAIDs, corticosteroids, anticoagulants, statins, non-SSRI antidepressants, antipsychotics, and clustering by unit setting
Eckert et al. (2024) [33]	Denmark	nested case control study	15,193	100	≥45	SSRI, TCA	8	Age at cohort entry, income quartiles, ischemic heart disease, atrial fibrillation, valvular disease, history of cancer, osteoporosis, diabetes, chronic obstructive pulmonary disease, history of lung cancer, loop diuretic use, systemic corticosteroids, SSRI, TCA, antipsychotics, opioids, statins, ASA, vitamin K antagonists, novel oral anticoagulants, insulin therapy, diuretics, renin-angiotensin inhibitors, and systemic HRT
Fu et al. (2022) [34]	USA	retrospective cohort study	13,831	53.2	<60	Not Classified	8	Age, sex, geographic region, insurance type, year at index, index VTE type, time from index VTE to treatment, comorbid conditions, concomitant medications, and procedures
Jick et al. (2008) [30]	UK	nested case control study	3867	65.2	≥18	SSRI, TCA, others	8	Smoking, BMI, OC, HRT, and antipsychotic agents
Lacut et al. (2007) [35]	France	case control study	1354	56.7	50–64	Not Classified	7	Age, gender, BMI, factor V Leiden, and prothrombin G20210A gene variation
Lee et al. (2015) [26]	Taiwan	retrospective cohort	105,822	61.6	15–59	SSRI, non-SSRI	8	Age, sex, AF, HTN, DM, CVA, HF, lower leg fracture or operations, and cancers
Marchena et al. (2020) [36]	Spain	retrospective cohort	49,007	49.5	63	Not Classified	8	Gender, chronic lung disease, anemia, cancer, transient risk factors for VTE, prior VTE, and initial VTE presentation (PE vs. DVT)
Parkin et al. (2003) [37]	New Zealand	case control study	375	68	≥65	Not Classified	9	Age, sex, weight, OC, and HRT
Parkin et al. (2017) [27]	UK	prospective cohort	734,092	100	≥65	SSRI, TCA, others	9	Age, BMI, smoking, alcohol consumption, frequency of strenuous physical activity, HTN, DM, high BP, and socioeconomic status, and stratified by recruitment region
Ray et al. (2002) [38]	Canada	retrospective cohort	131,196	63.5	≤70	SSRI, TCA	7	Sex, hospitalization, current residence within a long-term care facility, newly diagnosed cancer or concurrent prescription of either lithium or estrogen, and aspirin or warfarin
Schink et al. (2022) [39]	German	nested case control study	12,826	100	10–19	Not Classified	9	Age, other comorbidities, potential other (off-label) indications, lifestyle factors, and co-medication
Stuijver et al. (2013) [40]	Netherlands	population based case control study	21,297	57	17–73	Not Classified	8	Age, sex, hospitalizations (for malignancy, pregnancy, trauma, surgery, IBD, inflammatory arthritis, chronic respiratory failure), use of anticoagulants, drugs for respiratory diseases, antibiotics, antidepressants, and HRT and OC within 90 days prior to the index date
Svendsen et al. (2021) [41]	Denmark	case control study	68,696	100	≥55	Not Classified	8	Age, frequency of recurrent UTI, obesity, venous insufficiency, medication to treat cardiovascular disease, and history of VTE
Wu et al. (2013a) [29]	Taiwan	nested case control study	13,110	52.3	≥20	TCA, SSRI, SNRI, MAOI, NDRI, multiple users	8	Age, sex, and disease risk score
Wu et al. (2013b) [42]	Taiwan	case control study	15,128	51.8	≥16	Not Classified	9	CHD, HF, PVD, mood/psychotic disorders, hormones, lipid agents, mood stabilizers, antithrombotics, BZD, and clinic visits and hospitalizations prior 1 year
Zornberg et al. (2000) [43]	UK	population based nested case control study	214	76	≥18	Not Classified	8	Age, sex, general practice, index date, years of recorded history in the GPRD before the index date, BMI, smoking status, HTN, and estrogen use

AF, atrial fibrillation; ASA, acetylsalicylic acid (aspirin); BMI, body mass index; BP, blood pressure; BZD, benzodiazepine; CHD, coronary heart disease; CHF, congestive heart failure; CVA, cerebrovascular accident; DM, diabetes mellitus; DVT, deep vein thrombosis; GPRD, general practice research database; HF, heart failure; HRT, hormone replacement therapy; HTN, hypertension; IBD, inflammatory bowel disease; LD, liver disease; MAOI, monoamine oxidase inhibitor; MI, myocardial infarction; NDRI, norepinephrine-dopamine reuptake inhibitor; NSAIDs, nonsteroidal anti-inflammatory drugs; OC, oral contraceptive; PE, pulmonary embolism; PVD, peripheral vascular disease; SNRI, serotonin-norepinephrine reuptake inhibitor; SSRI, selective serotonin reuptake inhibitor; TCA, tricyclic antidepressant; UK, United Kingdom; USA, United States of America; UTI, urinary tract infection; VTE, venous thromboembolism.

## Data Availability

No new data were created or analyzed in this study. Data sharing is not applicable to this article.

## Supplementary Materials

The following supporting information can be downloaded at https://www.mdpi.com/article/10.3390/jcm14155512/s1: Supplementary Table S1. Newcastle–Ottawa Scale (NOS) Quality Assessment Scores for Included Studies; Supplementary Table S2. Summary of Data Sources and Collection Methods of Included Studies; Figure S1: Leave-one-out forest plot (with x-axis showing pooled relative risk per omission); Figure S2: Funnel plot for publication bias and short description of Begg/Egger test results. (cited references in supplementary material: 28–45)

## Abbreviations

The following abbreviations are used in this manuscript:

AF	atrial fibrillation
ASA	acetylsalicylic acid (aspirin)
BMI	body mass index
BP	blood pressure
BZD	benzodiazepine
CHD	coronary heart disease
CHF	congestive heart failure
CVA	cerebrovascular accident
DM	diabetes mellitus
DVT	deep vein thrombosis
GPRD	general practice research database
HF	heart failure
HRT	hormone replacement therapy
HTN	hypertension
IBD	inflammatory bowel disease
LD	liver disease
MAOI	monoamine oxidase inhibitor
MI	myocardial infarction
NDRI	norepinephrine-dopamine reuptake inhibitor
NSAIDs	nonsteroidal anti-inflammatory drugs
OC	oral contraceptive
PE	pulmonary embolism
PVD	peripheral vascular disease
SNRI	serotonin-norepinephrine reuptake inhibitor
SSRI	selective serotonin reuptake inhibitor
TCA	tricyclic antidepressant
UK	United Kingdom
USA	United States of America
UTI	urinary tract infection
VTE	venous thromboembolism.
