# Supplementary material for: Association Between Antidepressant Use and Risk of Venous Thromboembolism: A Systematic Review and Meta-Analysis
Source: J Clin Med. 2025 Aug 5;14(15):5512. doi: 10.3390/jcm14155512 (PMC12347909; doi:10.3390/jcm14155512)
Supplement: Supplementary file 1 [file jcm-14-05512-s001.zip › jcm-3781921-supplementary.pdf]

Supplementary Table S1. Newcastle–Ottawa Scale (NOS) Quality Assessment Scores for Included Studies

| Study name | Case-control | Is the case definition adequate? | Representative ness of the cases | Selection of Controls | Definition of Controls | Comparability of cases and controls on the basis of the design or analysis | Ascertainment of exposure | Same method of ascertainment for cases and controls | Non-Response rate | **Total** |
|---|---|---|---|---|---|---|---|---|---|---|
| | Cohort | Representative ness of the exposed cohort | Selection of the non-exposed cohort | Ascertainment of exposure | Demonstration that outcome of interest was not present at start of study | Comparability of cohorts on the basis of the design or analysis | Assessment of outcome | Was follow-up long enough for outcomes to occur | Adequacy of follow up of cohorts | |
| Aune et al (2024) [31] | Case-Crossover | **1** | **1** | **1** | **1** | **2** | **1** | **1** | **-** | **8** |
| Eckert et al (2024) [33] | Nested case control study | **1** | **1** | **1** | **1** | **2** | **1** | **1** | **-** | **8** |
| Jick et al (2008) [30] | Nested case-control study | **1** | **1** | **1** | **1** | **2** | **1** | **1** | **-** | **8** |
| Lacut et al (2007) [35] | Case-control study | **1** | **1** | **1** | **1** | **1** | **1** | **1** | **-** | **7** |
| Parkin et al (2003) [37] | Case-control study | **1** | **1** | **1** | **1** | **2** | **1** | **1** | **1** | **9** |
| Schink et al (2022) [39] | Nested case control study | **1** | **1** | **1** | **1** | **2** | **1** | **1** | **1** | **9** |
| Stuijver et al (2013) [40] | Population-Based Case-Control Study | **1** | **1** | **1** | **1** | **2** | **1** | **1** | **-** | **8** |

| | | | | | | | | | | |
|---|---|---|---|---|---|---|---|---|---|---|
| Svendsen et al (2021) [41] | Case control study | **1** | **1** | **1** | **1** | **2** | **1** | **1** | **-** | **8** |
| Wu et al (2013a) [29] | Nested case-control study | **1** | **1** | **1** | **1** | **2** | **1** | **1** | **-** | **8** |
| Wu et al (2013b) [42] | Case-control study cohort | **1** | **1** | **1** | **1** | **2** | **1** | **1** | **1** | **9** |
| Zornberg et al (2000) [43] | Population-Based Nested Case-Control Study | **1** | **1** | **1** | **1** | **2** | **1** | **1** | **-** | **8** |
| Bouyer et al (2018) [32] | Cohort | **1** | **1** | **1** | **1** | **2** | **1** | **1** | **-** | **8** |
| Bruun et al (2018) [28] | Retrospective Cohort | **1** | **1** | **1** | **1** | **2** | **1** | **-** | **1** | **8** |
| Fu et al (2022) [34] | Retrospective Cohort | **1** | **1** | **1** | **1** | **2** | **1** | **1** | **-** | **8** |
| Lee et al (2015) [26] | Retrospective Cohort | **1** | **1** | **1** | **1** | **2** | **1** | **1** | **-** | **8** |
| Marchena et al (2020) [36] | Retrospective Cohort | **1** | **1** | **1** | **1** | **2** | **1** | **1** | **-** | **8** |
| Parkin et al (2017) [27] | Prospective Cohort | **1** | **1** | **1** | **1** | **2** | **1** | **1** | **1** | **9** |
| Ray et al (2002) [38] | Retrospective Cohort | **1** | **1** | **1** | **1** | **1** | **1** | **1** | **-** | **7** |

Supplementary Table S2. Summary of Data Sources and Collection Methods of Included Studies

| Study name | Data Source | Data Type |
|---|---|---|
| Aune et al. (2024) [31] | Norwegian and Swedish national prescription databases | National registry |
| Bouyer et al. (2018) [32] | French nationwide hospital discharge database | Claims database |
| Bruun et al. (2018) [28] | Danish nationwide health registries | National cohort |
| Eckert et al. (2024) [33] | Hospital electronic records and national patient registry | Hospital-based + registry |
| Fu et al. (2022) [34] | U.S. administrative claims database | Claims database |
| Jick et al. (2008) [30] | UK General Practice Research Database (GPRD) | Primary care database |
| Lacut et al. (2007) [35] | French multicenter hospital cohort (EDITH study) | Hospital-based cohort |
| Lee et al. (2015) [26] | Taiwanese National Health Insurance Database (NHIRD) | Claims database |
| Marchena et al. (2020) [36] | RIETE registry (international registry for thromboembolism) | Clinical registry |
| Parkin et al. (2003) [37] | New Zealand mortality and prescription records | National population data |
| Parkin et al. (2017) [27] | Million Women Study (UK) | Large prospective cohort |
| Ray et al. (2002) [38] | U.S. Medicaid data (elderly population) | Claims database |
| Schink et al. (2022) [39] | German health insurance claims data | Claims database |
| Stuijver et al. (2013) [40] | Dutch PHARMO Record Linkage System | Linked cohort database |
| Svendsen et al. (2021) [41] | Danish national drug and patient registries | National registry |
| Wu et al. (2013a) [29] | Taiwan National Health Insurance Research Database | Claims database |
| Wu et al. (2013b) [42] | Taiwan National Health Insurance Research Database | Claims database |
| Zornberg et al. (2000) [43] | UK General Practice Research Database (GPRD) | Primary care database |

| Study name | Statistics with study removed | | | | |
|---|---|---|---|---|---|
| | Relative Risk | Lower limit | Upper limit | Z-Value | *p*-Value |
| Aune et al. 2024 | 1.204 | 1.103 | 1.314 | 4.145 | 0.000 |
| Bouyer et al. 2018 | 1.223 | 1.121 | 1.333 | 4.557 | 0.000 |
| Bruun et al. 2018 | 1.236 | 1.139 | 1.341 | 5.069 | 0.000 |
| Eckert et al. 2024 | 1.227 | 1.126 | 1.337 | 4.680 | 0.000 |
| Fu et al. 2022 | 1.205 | 1.109 | 1.310 | 4.389 | 0.000 |
| Jick et al. 2008 | 1.221 | 1.122 | 1.330 | 4.615 | 0.000 |
| Lacut et al. 2007 | 1.228 | 1.129 | 1.336 | 4.786 | 0.000 |
| Lee et al. 2015 | 1.232 | 1.137 | 1.336 | 5.069 | 0.000 |
| Marchena et al. 2020 | 1.227 | 1.129 | 1.333 | 4.808 | 0.000 |
| Parkin et al. 2003 | 1.212 | 1.118 | 1.314 | 4.666 | 0.000 |
| Parkin et al. 2017 | 1.204 | 1.105 | 1.311 | 4.265 | 0.000 |
| Ray et al. 2002 | 1.232 | 1.133 | 1.339 | 4.883 | 0.000 |
| Schink et al. 2022 | 1.206 | 1.111 | 1.309 | 4.478 | 0.000 |
| Stuijver et al. 2013 | 1.210 | 1.109 | 1.320 | 4.277 | 0.000 |
| Svendsen et al. 2021 | 1.210 | 1.095 | 1.336 | 3.743 | 0.000 |
| Wu et al. 2013a | 1.214 | 1.116 | 1.322 | 4.483 | 0.000 |
| Wu et al. 2013b | 1.181 | 1.107 | 1.259 | 5.036 | 0.000 |
| Zornberg et al. 2000 | 1.220 | 1.125 | 1.323 | 4.786 | 0.000 |
| | 1.216 | 1.121 | 1.319 | 4.730 | 0.000 |

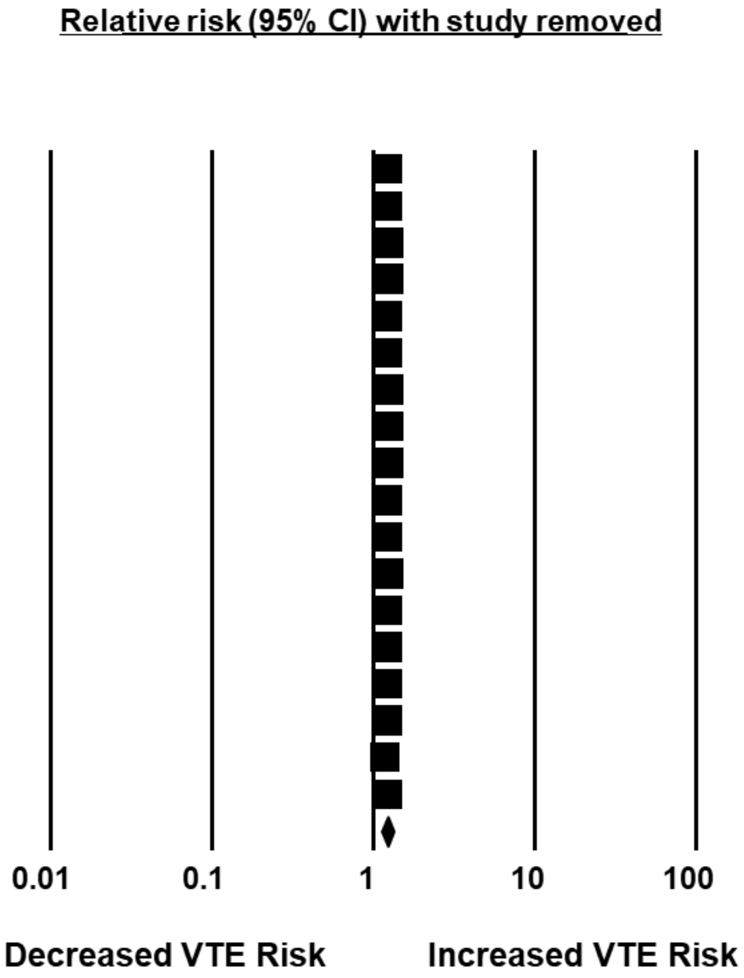


Figure S1: Leave-one-out forest plot (with x-axis showing pooled relative risk per omission)

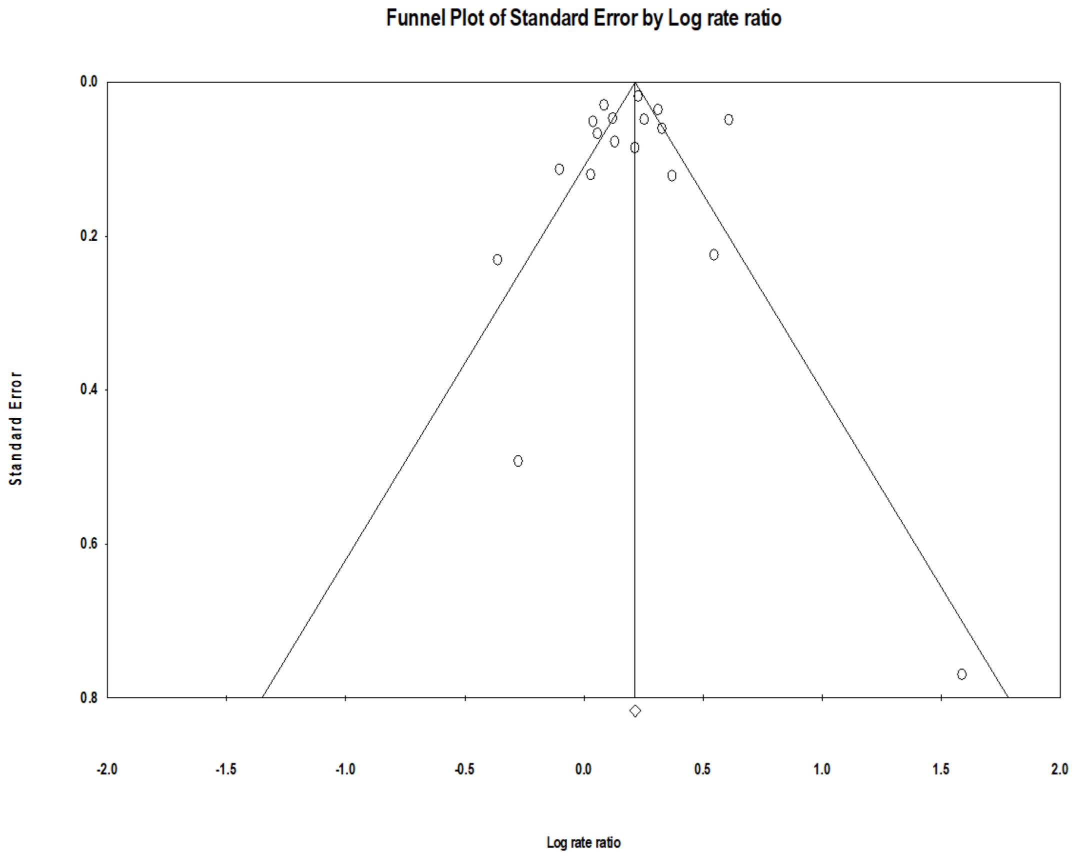


Begg rank correlation

Kendall's Tau
- tau 0.03922
- z-value for tau 0.2273
- p-value 0.82

Egger's regression intercept

- intercept -0.3061
- standard error 1.1374
- 95% lower limit -2.7172
- 95% upper limit 2.1050
- p-value 0.7913

Figure S2: Funnel plot for publication bias and short description of Begg/Egger test results
